# Molecular epidemiology of methicillin-resistant *Staphylococcus aureus* in Gulf Cooperation Council countries (2010–2025): a scoping review

**DOI:** 10.3389/fmicb.2025.1714252

**Published:** 2025-11-19

**Authors:** Ahmed Briki, Nada Alkhatib, Bisola Aloba, Subham Verma, Rania Nassar, Dean Everett, Ralf Ehricht, Stefan Monecke, Abiola Senok

**Affiliations:** 1 College of Medicine, Mohammed Bin Rashid University of Medicine and Health Sciences, Dubai Health, Dubai, United Arab Emirates; 2 Department of Public Health and Epidemiology, College of Medicine and Health Sciences, Khalifa University, Abu Dhabi, United Arab Emirates; 3 Infection Research Unit, Khalifa University, Abu Dhabi, United Arab Emirates; 4 Leibniz Institute of Photonic Technology (IPHT), Leibniz Center for Photonics in Infection Research (LPI), Jena, Germany; 5 InfectoGnostics Research Campus, Jena, Germany; 6 Institute of Physical Chemistry, Friedrich-Schiller University, Jena, Germany; 7 School of Dentistry, Cardiff University, Cardiff, United Kingdom

**Keywords:** *S. aureus*, methicillin-resistant, antimicrobial resistance, epidemiology, prevalence, GCC, Middle East

## Abstract

**Background:**

Methicillin-resistant *Staphylococcus aureus* (MRSA) is a global public health concern, including within the Gulf Cooperation Council (GCC). As an opportunistic pathogen, MRSA poses a threat to hospitalized patients, and to the wider community. Its success is largely attributed to its diverse virulence factors and adaptability, with antimicrobial resistance further enhancing its persistence and complicating treatment efforts. In the GCC, the epidemiology of MRSA is influenced by several distinctive factors, including the region’s diverse demographics, high population mobility, and variations in healthcare infrastructure. Understanding the molecular epidemiology is crucial to curb transmission and guide effective public health measures.

**Aim:**

This scoping review evaluates MRSA data in GCC countries—United Arab Emirates (UAE), Saudi Arabia, Kuwait, Qatar, Oman, and Bahrain—focusing on prevalence trends, molecular characteristics, and gaps in the literature.

**Methods:**

A systematic search of the PubMed (National Library of Medicine [NLM], National Institutes of Health [NIH]) database was conducted to identify literature published between January 2010 and July 2025, using predefined keywords on MRSA epidemiology. Eligible studies were reviewed for MRSA prevalence, clonal diversity, antimicrobial resistance patterns, and virulence profiles.

**Results:**

Of 864 records screened, 97 met the inclusion criteria and were subjected to detailed review. Most studies originated from Saudi Arabia (58%), followed by Kuwait (26%), UAE (7%), Oman (3%), Qatar (2%), Bahrain (2%), and two involving adjacent countries (2%). Data indicate a predominance of community-associated MRSA (CA-MRSA) across both healthcare and community settings. Several studies reported novel or rare MRSA strains across various GCC countries. Moreover, there is clear evidence of widespread resistance to multiple classes of antibiotics, highlighting the growing concern of multidrug-resistant MRSA. Increasing prevalence of strains with virulence/resistance genes such as *pvl* and SCC*mec*+f*usC*, associated with enhanced pathogenicity and resistance, was also observed. Along with wide clonal diversity, frequent travel, and differing infection control practices contribute to the region’s complex MRSA epidemiology.

**Conclusion:**

MRSA in the GCC shows dynamic and evolving patterns. Continuous surveillance through coordinated regional efforts is essential. A One Health approach, combined with strengthened antimicrobial stewardship, mandatory hospital screenings, and wastewater monitoring, could improve MRSA detection, tracking, and control across the region.

## 1 Introduction

*Staphylococcus aureus* is a Gram-positive bacterium that forms part of the normal microbiota in 20–30% of the healthy human population (Laux et al., 2019; Kluytmans et al., 1997). While often a harmless commensal, it can also act as an opportunistic pathogen associated with a wide spectrum of infections, from superficial skin and soft tissue infections to infective endocarditis, life-threatening sepsis and toxic shock syndrome (Shoaib et al., 2022). Patients with invasive medical devices or compromised immune systems are particularly vulnerable to *S. aureus* infections (Lowy, 1998). The effectiveness of penicillin as drug of choice to combat *S. aureus* infections was short-lived as resistance emerged by the mid-1940s, only a few years after its introduction into clinical use (Lowy, 1998). Whilst methicillin was developed as an alternative, there was a rapid emergence of methicillin-resistant *Staphylococcus aureus* (MRSA) with the first report in the early 1960s in the United Kingdom (Enright et al., 2002). Beta-lactam antibiotics act on penicillin-binding protein (PBP), an enzyme that catalyzes the cross-linking of the subunits constituting the bacteria’s peptidoglycan cell wall (Mora-Ochomogo and Lohans, 2021). MRSA resistance occurs via acquisition of the *mecA* gene which encodes for a modified penicillin-binding protein called PBP2a, that has a reduced binding affinity for beta-lactam antibiotics, rendering them ineffective (Pinho et al., 2001). In addition to *mecA*, other *mec* homologs (*mecB*, *mecC*, and *mecD*) have also been described, each encoding alternative PBPs that confer similar *β*-lactam resistance phenotypes, though they are less frequently detected in clinical isolates (González-Machado et al., 2024). Asymptomatic carriage of MRSA not only represents a major risk factor for invasive infection but also facilitates transmission within both healthcare and community settings (Azzam et al., 2025). With its widespread global dissemination and the shifting molecular epidemiology landscape, MRSA remains a priority antimicrobial resistance (AMR) pathogen and a significant public health threat (Azzam et al., 2025).

In a recent assessment of the global AMR burden, MRSA demonstrated the largest increase in both deaths attributable to and deaths associated with, AMR, making it a pathogen of great concern (GBD 2021 Antimicrobial Resistance Collaborators, 2024). In terms of attributable deaths, MRSA was responsible for approximately 130,000 deaths worldwide in 2021, which is more than double the 57,200 deaths reported in 1990 (GBD 2021 Antimicrobial Resistance Collaborators, 2024). The number of deaths associated with MRSA increased globally from 261,000 deaths to 550,000 over the same period (GBD 2021 Antimicrobial Resistance Collaborators, 2024). On a regional scale, trends in the United Arab Emirates (UAE) also demonstrate increased prevalence of MRSA (Thomsen et al., 2023). AMR surveillance data revealed that a total of 29,414 MRSA isolates were reported in the UAE between 2010 and 2021, accounting for 26.4% of all *S. aureus* isolates identified during this 11-year period (Thomsen et al., 2023). An upward annual trend was observed, with MRSA comprising 21.9% (259/1,181) of *S. aureus* isolates in 2010, increasing to 33.5% (4,996/14,925) by 2021 (Thomsen et al., 2023). Analysis from a large tertiary care hospital in Saudi Arabia between 2006 and 2015 reported an overall annual MRSA incidence rate of 25 cases per 100,000 patients, accounting for 27% of all *S. aureus* isolates (Al-Hamad et al., 2018). More recent studies from Saudi Arabia have reported persistently high MRSA prevalence rates, ranging from 45.4 to 54.8% (Almutairi et al., 2024; Alhazmi et al., 2025). Across the Gulf Cooperation Council (GCC) region, MRSA prevalence varies considerably between countries (Alhazmi et al., 2025; Khawaja et al., 2021; Senok et al., 2020; Boucherabine et al., 2025; Tabaja et al., 2021; Alfouzan et al., 2023; El-Mahdy et al., 2014; Alarawi et al., 2025). Nonetheless, an overall upward trend in MRSA incidence has been documented throughout the GCC (Alhazmi et al., 2025; Khawaja et al., 2021; Senok et al., 2020; Boucherabine et al., 2025; Tabaja et al., 2021; Alfouzan et al., 2023; El-Mahdy et al., 2014; Alarawi et al., 2025; Al-Saleh et al., 2022), and in countries with strong epidemiological links to the region, largely through work-related migration (Tabaja et al., 2021; Patil et al., 2022).

Despite considerable efforts to control its spread, *S. aureus*, particularly MRSA, continues to persist, in part due to its ability to acquire resistance not only to methicillin and other broad-spectrum antibiotics, but also to topical decolonizing agents such as mupirocin and fusidic acid (Hajhamed et al., 2025). Beyond its multi-drug resistance, MRSA also harbors a wide arsenal of virulence factors that contribute to its pathogenicity (Howden et al., 2023). Among these, the cytotoxic pore-forming Panton-Valentine leukocidin (PVL) toxin has garnered significant attention for its potential role in targeting, penetrating and destroying leukocytes, which leads to tissue damage and necrosis (Gulmez et al., 2012). Globally, the PVL toxin is often associated with epidemic community-associated MRSA (CA-MRSA) strains, which are implicated in a range of severe infections, including skin and soft tissue infections, necrotizing pneumonia, and fatal cases of necrotizing fasciitis (Boyle-Vavra and Daum, 2007).

The Staphylococcal Chromosomal Cassette *mec* (SCC*mec*) mobile genetic element, which harbors the *mecA* or *mecC* gene, responsible for methicillin resistance has also been extensively studied (Katayama et al., 2000). The structural diversity of SCC*mec* elements across different MRSA strains has significant implications for the pathogen’s evolution, transmission patterns, and classification into healthcare-associated or community-associated lineages (Deurenberg and Stobberingh, 2008), making it a focal point in both clinical and molecular studies of MRSA. Historically, the type of SCC*mec* element present often correlated with the epidemiological origin of the strain; types I–III with healthcare-associated MRSA lineages (HA-MRSA), and type IV/V with CA-MRSA (Deurenberg and Stobberingh, 2008).

Nowadays, the HA-MRSA/CA-MRSA traditional grouping of MRSA is losing relevance and is now largely only of historical value (Vestergaard et al., 2019). Definitions and criteria for distinguishing CA-MRSA from HA-MRSA often also vary between studies, further complicating interpretation (Kateete et al., 2019). The distinction is becoming progressively blurred as CA-MRSA strains are increasingly identified in healthcare settings as drivers of nosocomial infections and HA-MRSA strains detected in the community (Ito et al., 2004). Globally, the dissemination of MRSA has been shaped by several well-characterized clonal lineages, including Epidemic MRSA-15 (EMRSA-15/ST22-MRSA-IV) (Turner et al., 2019), which predominates in Europe; USA300 (ST8-MRSA-IV) (Nimmo, 2012), the major community-associated clone originating in North America; and ST239, a long-standing hospital-associated lineage prevalent across Asia and the Middle East (Monecke et al., 2018). Other internationally recognized clones, such as ST80 in Europe and North Africa and ST772-MRSA-V (“Bengal Bay clone”) in the Indian subcontinent, further highlight the geographic structuring and adaptive diversity of MRSA populations (Otter and French, 2012; Steinig et al., 2022). In the GCC region for instance, many dominant strains carry both *mecA* and the fusidic acid resistance (*fusC*) gene on a single SCC*mec* element and may also harbor PVL (Boucherabine et al., 2025). These strains possess a dual advantage whereby they thrive in nosocomial settings due to methicillin resistance, and they also maintain a selective advantage in the community where fusidic acid use is high (Baines et al., 2016). Widespread use of antimicrobial agents, and subsequent acquisition of resistance mechanisms, has contributed to the generation of selective pressure and expansion of epidemic CA-MRSA clones such as the globally recognized PVL-positive USA300 (CC8/ST8-MRSA-IV) (Planet et al., 2013).

Consequently, this makes traditional HA/CA classifications increasingly redundant. As the epidemiology of MRSA continues to shift, recognizing this dual ecological fitness is crucial for conducting more accurate and meaningful epidemiological investigations. The shifts in MRSA’s landscape and its designation as a priority pathogen by the World Health Organization (WHO) underline the critical need for continuous surveillance and monitoring to effectively address the evolving threat posed by MRSA (Tkadlec et al., 2023; Robinson and Enright, 2004).

The Gulf Cooperation Council (GCC) region, which comprises the UAE, the Kingdom of Saudi Arabia (KSA), Oman, Qatar, Kuwait, and Bahrain, spans a vast geographical area and is characterized by a highly dynamic and demographically diverse population. The population dynamics are largely shaped by several contributing factors, including substantial immigration, mass gathering events including annual pilgrimages in Saudi Arabia, frequent international travel, and a growing trend of medical tourism across the region (Memish and Shibl, 2011). This unique pattern of population movement within the GCC landscape greatly facilitates the introduction, circulation and evolution of infectious diseases, including those caused by multidrug-resistant (MDR) pathogens (Memish and Shibl, 2011). As a result, the GCC region presents a complex and varied pattern of AMR, with MRSA representing a particularly significant public health concern. The molecular landscape of MRSA in the GCC is characterized by a wide clonal diversity, varying resistance mechanisms, and the emergence of both local and globally disseminated strains, highlighting the need for coordinated regional surveillance, research, and control strategies.

While surveillance data and infection prevention and control guidelines are available, genomic data in the GCC countries remains fragmented and largely unstructured, limiting their utility for comprehensive analysis and surveillance. Genomic data provides a robust foundation for a better understanding of prevalence, uncovering transmission dynamics and dissemination patterns, as well as informing development of more effective control strategies (Sundermann et al., 2025). To this end, this review aims to provide a comprehensive overview of the literature on the molecular epidemiology and genomic characterization of MRSA isolates circulating in the countries of the GCC region. In addition, this scoping review also maps emerging trends, settings of infection, predominant population groups as well as knowledge gaps in the literature which warrant further investigation and potential strategies to address some of the driving factors behind rising MRSA resistance in the region. Reviews of this nature are critical for guiding the development of evidence-based infection prevention and control policies, antimicrobial stewardship programs, and region-specific research strategies.

## 2 Methods

This review was conducted following an established methodology for scoping studies, as previously described by Arksey and O'malley (2005). The review process comprised of five key stages: (i) identification of the research question, (ii) identification of relevant studies, (iii) study selection (iv) charting the data, and (v) collating, summarizing, and reporting the results. The Preferred Reporting Items for Systematic Reviews and Meta-Analyses (PRISMA) checklist was used to guide and document the review process.

### 2.1 Search strategy

A comprehensive search was conducted in PubMed (National Library of Medicine [NLM], National Institutes of Health [NIH]) to identify studies on MRSA in the GCC. Search terms included “MRSA,” “Methicillin-Resistant *Staphylococcus aureus*,” “UAE,” “Saudi Arabia,” “Oman,” “Kuwait,” “Qatar,” “Bahrain,” “Gulf Region,” and “GCC,” with Boolean operators applied where appropriate. The precise search strategy is provided with Supplementary Table 1.

PubMed captured all peer-reviewed journal articles indexed in MEDLINE, as well as additional records from PubMed Central and online-first publications. It was selected as the primary database due to its extensive coverage of biomedical and life sciences literature and its inclusion of most regional and international journals publishing MRSA-related studies. Regional journals containing relevant studies were also captured through our searches in Scopus and PubMed, which index the majority of such journals. Preliminary scoping searches in Scopus confirmed that all relevant literature on MRSA molecular typing in the GCC were already indexed in PubMed. The search encompassed publications from January 1, 2010, to July 31, 2025, with the final search conducted on September 8, 2025.

### 2.2 Selection criteria

Two independent reviewers screened titles, abstracts, and full texts for eligibility using a standardized data extraction spreadsheet; no automated screening software was employed. Discrepancies were resolved through discussion or, if necessary, arbitration by a third reviewer. As this was a scoping review, a formal quality appraisal of included studies was not conducted. The inclusion criteria comprised original research studies on MRSA conducted in any GCC country and published in English. Studies were excluded if they were case reports, case series, editorials, letters, systematic reviews, focused on treatments or general epidemiology, or conducted in the GCC but primarily reporting on non-GCC populations, ensuring the review specifically addressed the molecular epidemiology of MRSA.

For each included study, data were extracted on the country, study period, study design, sample size, and population or setting (e.g., hospital, community, food supply). Key findings were also recorded, including the detection of PVL, resistance genes (e.g., *mecA*, *fusC*, *vanA*), and reported phenotypic resistance to commonly used antibiotics. A breakdown of the key terms focused on during the data extraction process from the reviewed papers is provided (Table 1). Additionally, notable observations such as the first identification of specific clones or documented outbreak events were systematically captured.

**Table 1:** Key MRSA-related terms emphasized during data extraction from the reviewed studies.

Term	Definition*
MLST	Multilocus sequence typing: Sequence-based method that characterizes isolates by sequencing fragments of typically seven housekeeping genes to assign sequence types (STs).
CC	Clonal complex: Group of related MLSTs that share identical alleles at most loci, indicating a common evolutionary origin.
ST	Sequence type: Numerical genotype assigned through MLST based on the allelic profile of housekeeping genes.
*spa* typing	Typing method based on sequence variation in the polymorphic X-region of the staphylococcal protein A *(spa)* gene; *spa* types are designated as *t#* (e.g., t037).
SCC*mec*	Staphylococcal cassette chromosome *mec*: Mobile genetic element carrying *mec* genes that confer *β*-lactam resistance; classified into types I–XV based on structure and gene content.
*mecA*	Gene encoding penicillin-binding protein 2a (PBP2a), which has low affinity for β-lactam antibiotics, conferring methicillin resistance.
*mecC*	Homolog of *mecA* that encodes a PBP2a-like protein; less common and often associated with animal or environmental isolates.
PVL	Panton–Valentine leukocidin: Genes (*lukS-PV* and *lukF-PV*) encoding a bicomponent pore-forming leukotoxin associated with severe skin and soft tissue infections.
*fusC*	Gene conferring fusidic acid resistance, often located on SCC elements.
*fusA*	Chromosomal gene encoding elongation factor G (EF-G); specific mutations mediate high-level fusidic acid resistance.
*fusB*	Plasmid- or transposon-borne fusidic acid resistance determinant that protects EF-G, distinct from *fusC*-mediated resistance.
*vanA*/*vanB*	Operons encoding altered cell wall precursors that confer glycopeptide (vancomycin/teicoplanin) resistance; *vanA* typically confers high-level resistance.
*vraA* (and related mutations)	Genes involved in the *vraSR*-mediated cell wall stress response pathway; mutations are associated with intermediate vancomycin resistance (VISA) or elevated minimum inhibitory concentrations (MICs).
*cfr*	Gene encoding a 23S rRNA methyltransferase that confers resistance to linezolid and other phenicols, lincosamides, oxazolidinones, pleuromutilins, and streptogramin A antibiotics.
*qac* genes	Genes encoding multidrug efflux pumps that reduce susceptibility to antiseptics such as chlorhexidine and quaternary ammonium compounds.
ACME	Arginine catabolic mobile element: Genomic island, often in USA300, that enhances skin colonization and bacterial fitness.
*tst* (TSST-1)	Gene encoding toxic shock syndrome toxin-1, a superantigen implicated in toxic shock syndrome.
*sea*, *seb*, *sek*, seq	Genes encoding staphylococcal enterotoxins and related superantigens associated with food poisoning and immune activation.
CA-MRSA	Community-associated MRSA: MRSA acquired in the community, often carrying SCC*mec* types IV or V and *PVL*-positive lineages.
HA-MRSA	Healthcare-associated MRSA: MRSA acquired in healthcare settings; typically carries SCC*mec* types I–III and exhibits multidrug resistance.
LA-MRSA	Livestock-associated MRSA: MRSA lineages adapted to animals and animal products, most notably ST398.
MDR	Multidrug-resistant: Non-susceptible to at least one agent in ≥3 antimicrobial classes (CDC definition).
MIC creep	Gradual increase in minimum inhibitory concentration (MIC) values within the susceptible range over time, suggesting emerging resistance.
WGS	Whole-genome sequencing: High-resolution sequencing of the complete bacterial genome for genotyping, profiling resistance/virulence determinants and phylogenetic analysis.
DNA microarray	High-throughput hybridization assay for simultaneous detection of multiple resistance, virulence, or clonal markers.
PCR	Polymerase chain reaction: Targeted amplification of specific DNA sequences for detection or characterization of genes.
AMR genes	Antimicrobial resistance genes: Genes conferring resistance to one or more antimicrobial agents.
Virulence genes	Genes encoding factors that promote infection, disease progression, or immune evasion.
E-MRSA	Epidemic MRSA: MRSA strains causing outbreaks among multiple patients across hospitals or regions (epidemiological designation).

* Definitions compiled from authoritative sources, including the Centers for Disease Control and Prevention (CDC), and peer-reviewed literature retrieved from the PubMed database.

## 3 Results

**Figure 1:**
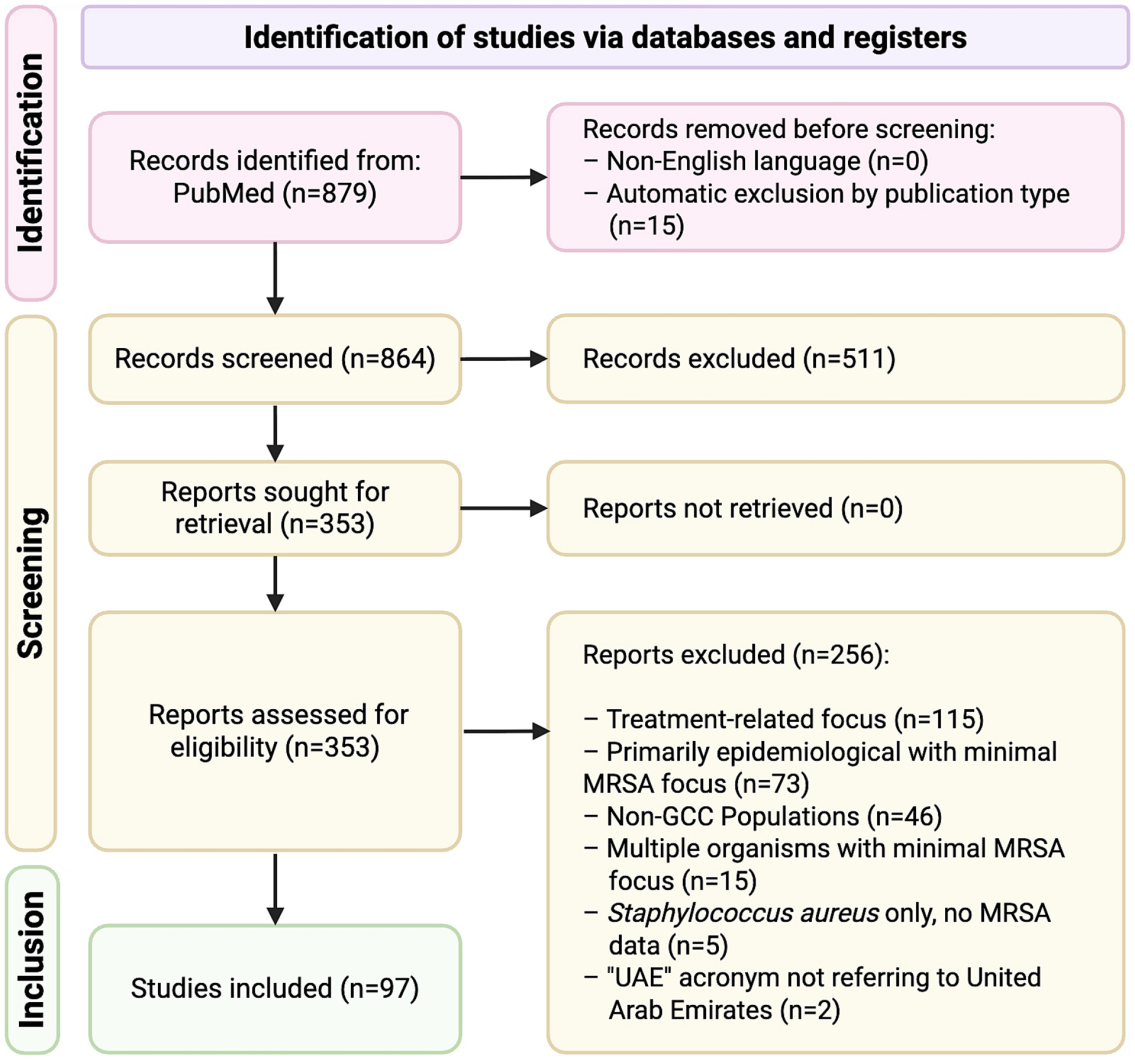
Preferred reporting items for systematic reviews and meta-analyses (PRISMA) flow diagram of article selection process. Covered peer-reviewed journal articles indexed in MEDLINE on PubMed (National Library of Medicine [NLM], National Institutes of Health [NIH]), records from PubMed Central and online-first publications between January 1, 2010 – July 31, 2025.

**Figure 2:**
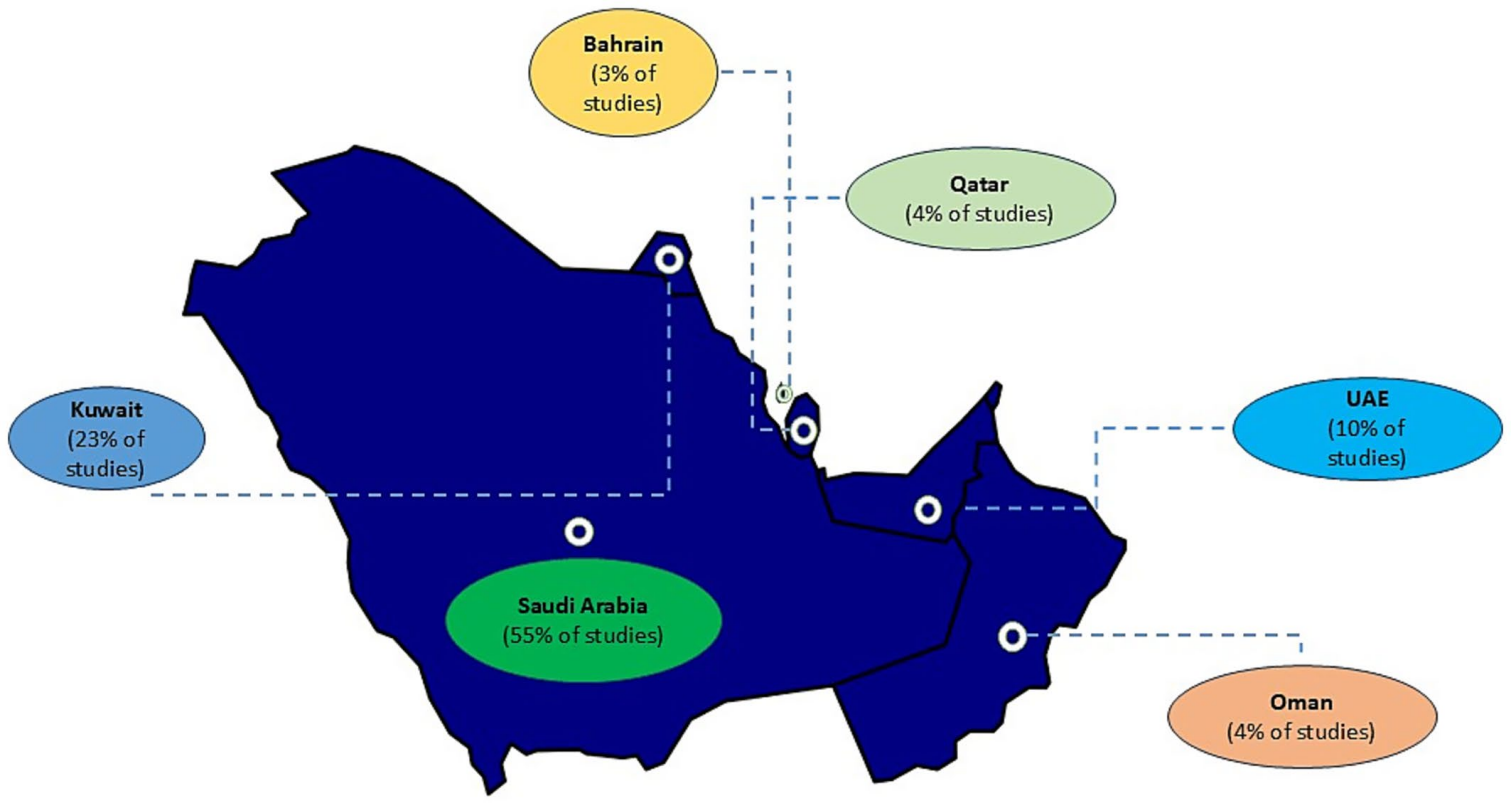
Distribution of MRSA studies across the Gulf Cooperation Council (GCC) region (January 2010–July 2025). Shown are the studies that met the inclusion criteria for this review, illustrating the uneven distribution and regional disparities in published MRSA research output. Studies were conducted in Saudi Arabia (*n* = 56), Kuwait (*n* = 25), and the United Arab Emirates (*n* = 7), with two additional studies including data from all three countries (2%). Limited literature was available for Bahrain (*n* = 3), Oman (*n* = 2), and Qatar (*n* = 2). Most studies were hospital- or tertiary care–based (*n* = 76).

A total of 864 records were screened following the initial search. After screening and applying inclusion/exclusion criteria, 97 studies were included in the final review (Supplementary Table 1). A PRISMA flow diagram illustrating the study selection process is provided (Figure 1). The majority of studies were conducted in Saudi Arabia (*n* = 56, 58%), followed by Kuwait (*n* = 25, 26%) and the United Arab Emirates (*n* = 7, 7%), with two additional studies including data from all three countries (2%) (Figure 2). In contrast, only a limited number of studies were available for Bahrain (*n* = 3, 3%), Oman (*n* = 2, 2%) and Qatar (*n* = 2, 2%) (Figure 2). Most studies were hospital- or tertiary care–based (*n* = 76). In addition, ten studies investigated MRSA within the food supply chain (e.g., raw meat, milk), and two focused on livestock. With the exception of one study from the UAE and one from Qatar, all food- and livestock-related investigations originated from Saudi Arabia.

Regarding molecular typing approaches, PCR-based methods were the most commonly employed, used in 31 studies across the GCC. DNA microarray–based typing was utilized in eleven studies, and whole-genome sequencing (WGS) was reported in five studies, reflecting its growing but still limited use in the region (Supplementary Table 1). An additional 18 studies applied a combination of molecular techniques to enhance resolution and confirm clonal relationships, while the remaining 32 studies did not perform molecular typing, relying instead on phenotypic or antimicrobial susceptibility data (Supplementary Table 1).

**Figure 3:**
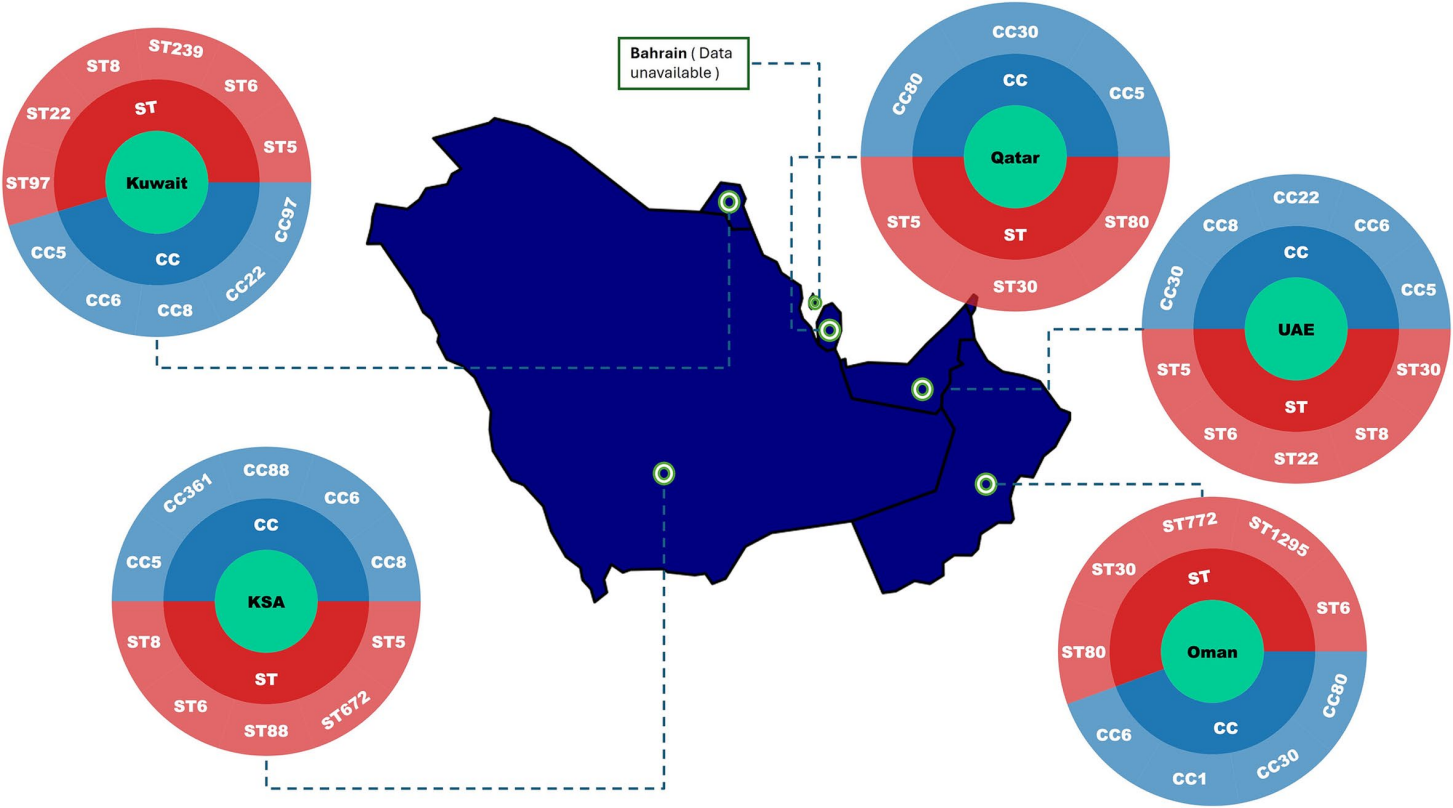
Distribution of predominant MRSA clonal complexes (CCs) and sequence types (STs) across the Gulf Cooperation Council (GCC) region (January 2010–July 2025). The map highlights the main MRSA lineages reported in included studies. Studies were conducted in Saudi Arabia (*n* = 56), Kuwait (*n* = 25), and the United Arab Emirates (*n* = 7), with two additional studies including data from all three countries (2%). Limited literature was available for Bahrain (*n* = 3), Oman (*n* = 2), and Qatar (*n* = 2). Country with limited molecular data are indicated accordingly. Abbreviation: UAE, United Arab Emirates.

Between 2010 and 2025, the epidemiology of MRSA across the GCC countries has shown shared regional patterns alongside notable country-specific differences (Figure 3; Table 2). Multiple clonal complexes (CCs) are shared across the region, with CC5, CC22, CC80, CC1, CC30, and CC6 being the most frequently identified (Figure 3) (Senok et al., 2020; El-Mahdy et al., 2014; Boswihi et al., 2018). Data from the UAE, Kuwait, Saudi Arabia, Qatar, Oman, and Bahrain indicate a gradual epidemiological shift toward the predominance of CA-MRSA over the last decade (Senok et al., 2020; Boswihi et al., 2018; Boswihi et al., 2016). In numerous recent hospital-based studies, the majority of MRSA isolates carry SCC*mec* types IV or V, which are typically associated with CA-MRSA phenotypes (Senok et al., 2020; Boswihi et al., 2020; Udo et al., 2014; Alsaleh et al., 2023), although SCC*mec* types I–III persist in smaller proportions (Boswihi et al., 2018; Boswihi et al., 2020; Udo et al., 2014). Traditional HA-MRSA clones such as sequence type (ST)-239 remain present (El-Mahdy et al., 2014; Boswihi et al., 2018; Boswihi et al., 2020), although they are increasingly declining and being outnumbered by CA-MRSA genotypes. Reports of fusidic acid resistance linked to *fusC*-bearing SCC*mec* elements are increasingly common in several countries (Senok et al., 2020; Boswihi et al., 2020; Senok et al., 2019a). This trend likely reflects widespread community use of topical fusidic acid, which in some settings is available over-the-counter. Topical fusidic acid is inexpensive and easily accessible. High prevalence of PVL-positive MRSA in the GCC may also drive frequent fusidic acid use, as these strains are associated with skin and soft tissue infections, further promoting selection for resistance (Bourles et al., 2022). Carriage of *pvl* genes is common across the region, though prevalence varies substantially, typically ranging from 20 to 50% in large hospital studies and occasionally exceeding these percentages (Senok et al., 2020; El-Mahdy et al., 2014; Udo et al., 2014; Alsaleh et al., 2023).

**Table 2:** Summary of key findings on MRSA epidemiology in GCC countries.

Country	Key findings	Key resistance & virulence determinants	Notable observations/ emerging trends
United Arab Emirates	MRSA prevalence rose 21.9% (2010) → 33.5% (2021) Increasing clonal diversity Predominantly CA-MRSA (SCC*mec* IV/V)	*pvl* (up to 49%) SCC*mec*-*fusC* (14–28.9%) Low linezolid resistance (≤2.9%), no VRSA detected	First reports: PVL + CC398, CC5-MRSA-IV “Sri Lanka clone” MRSA in food chain CA-MRSA increasingly hospital-acquired
Saudi Arabia	Shift to CA-MRSA dominance Increasing clonal diversity SCC*mec* IV/V/VI/VII Nationwide genomic surveillance performed (2022–2024)	*pvl* (30–50%) *fusC* (up to 46%), aminoglycoside, fluoroquinolone resistance genes common Low linezolid resistance (2.9%), *cfr* gene detected Vancomycin-intermediate strains & VRSA (vanA+) reported	Cross-border clone spread (CC361) Zoonotic/foodborne MRSA (camel, goat, dairy products) Rapid expansion of USA300/ST8 subclade
Kuwait	High circulation of HA-MRSA and CA-MRSA lineages Persistence of ST239-III Gradual shift to diverse CA-MRSA clones SCC*mec* III/IV/V	*pvl* (up to 45%) Fusidic acid resistance (~80%) (*fusC*/*fusB*/*fusA*) Chloramphenicol resistance (fexA+ CC5) Rising vancomycin MICs	Emergence & expansion of CC361-MRSA [V/VT + fusC] Local evolution of CC239 variant Limited food/animal MRSA data
Oman	CA-MRSA predominates High SCC*mec* IV carriage	*pvl* (44.3%) Growing prevalence of MDR	Emerging MDR ST22-IV/t852 variant
Qatar	CA-MRSA predominates SCC*mec* IV/V Limited molecular data	PVL-positive USA1100 “Southwest Pacific Clone” reported *pvl/fusC* (Data unavailable) Mupirocin resistance noted	CA-MRSA dominance mirrors regional/global trends.
Bahrain	Limited molecular data SCC*mec* IV/V Recent study: 88% CA-MRSA, 22% MDR	*pvl* (66% of CA-MRSA, 33% of HA-MRSA) *fusC* (Data unavailable) MDR associated with quinolones, macrolides, folate antagonists.	PVL prevalence higher than neighboring GCC countries Bahrain highlights the urgent need for comprehensive surveillance in all countries

Raw camel meat, dairy products, milk and goats. CA-MRSA, Community-associated methicillin-resistant *Staphylococcus aureus*; HA-MRSA, Healthcare-associated MRSA; PVL, Panton–Valentine leukocidin; fusC, fusidic acid resistance gene; MDR, Multidrug-resistant; SCCmec, Staphylococcal cassette chromosome mec; GCC, Gulf Cooperation Council.

From an antimicrobial resistance perspective, MRSA remains universally resistant to *β*-lactam antibiotics. However, resistance to non-β-lactam agents is a growing concern. High rates of fusidic acid resistance, frequent resistance to macrolides and fluoroquinolones, and variable rates of aminoglycoside resistance are consistently documented across the GCC (Senok et al., 2020; Boswihi et al., 2018; Boswihi et al., 2020). Glycopeptides and oxazolidinones, specifically vancomycin and linezolid, continue to demonstrate good *in vitro* activity (Senok et al., 2020; El-Mahdy et al., 2014; Udo et al., 2014). Nonetheless, “vancomycin minimum inhibitory concentration (MIC) creep” has been reported, and isolated cases of linezolid non-susceptibility have emerged (Boswihi et al., 2020; Aljohani et al., 2020; Al-Sarar et al., 2024). Vancomycin resistance in MRSA remains rare; however, cases have been reported in Saudi Arabia, including isolates harboring the *vanA* gene in one study and mutations in the *vraR* response regulator gene in another (Al-Sarar et al., 2024; Almuhayawi et al., 2023; Alhejaili et al., 2025). The limited emergence of resistance may reflect the restricted use of vancomycin and linezolid, which is constrained by both their pharmacokinetic considerations and high cost, limiting widespread or inappropriate administration (Alghanem et al., 2023; Rao et al., 2020).

The majority of available MRSA data in the GCC originate from hospital-based surveillance, targeted screening programs, or outbreak investigations, with limited longitudinal, population-level surveillance. Inter-country comparisons are further complicated by differences in study methodologies, including variation in laboratory diagnostic techniques, and sampling strategies. Overall, DNA microarray-based typing technology is the most frequently employed technique for genotypic profiling, clonal typing, and surveillance of MRSA across the GCC (Senok et al., 2020; Boswihi et al., 2018; Boswihi et al., 2020; Alfouzan et al., 2019; Senok et al., 2018; Udo et al., 2020). Despite increasing availability of whole-genome sequencing technologies, DNA microarray platforms remain the preferred approach, potentially reflecting considerations related to cost, ease of interpretation, and scalability (Deurenberg and Stobberingh, 2008; Shore et al., 2012; El Garch et al., 2009).

### 3.1 MRSA in the UAE

Available data in the UAE suggests a high burden of MRSA, growing prevalence and increasing diversity in the clonal complexes (CCs) circulating in the country. Most notably, the proportion of MRSA isolates reported increased significantly from 21.9% in 2010 to 33.5% in 2021 (Thomsen et al., 2023), reflecting a concerning upward trend in resistance and the persistence of MRSA. This 12-year retrospective analysis reported on 29,414 clinical MRSA isolates identified as part of the UAE national AMR surveillance program (Thomsen et al., 2023). The study revealed rising resistance trends to several antibiotics, including ciprofloxacin, levofloxacin, moxifloxacin, erythromycin, gentamicin, trimethoprim-sulfamethoxazole, and quinupristin/dalfopristin (Thomsen et al., 2023). Linezolid resistance remained low (0.0–0.8%) throughout most of the study period, with slightly higher rates observed in 2015 (2.5%), 2016 (2.6%), and 2017 (2.9%). Notably, no confirmed cases of vancomycin-resistant *S. aureus* were reported (Thomsen et al., 2023).

A study that genotyped 625 clinical MRSA isolates from across three Emirates (Dubai, Abu Dhabi and Umm Al Quwain) identified 23 clonal complexes and 102 strains with CC5, CC6, CC22 and CC30 as the most prevalent, collectively accounting for 54.2% of the isolates (Senok et al., 2020). Other common CCs identified were CC1, CC8, CC772, CC361, CC80, and CC88. Many of the strains belonged to CA-MRSA lineages harboring SCC*mec* types IV and V (Senok et al., 2020). In addition, this report documented the identification of several globally recognized MRSA clones including the ACME-negative/PVL-positive CC8-MRSA-[SCC*mec* IVc + Hg], CC22-MRSA-Iva “UK EMRSA-15/Barnim Epidemic Clone,” CC772-MRSA-V (PVL+) “Bengal-Bay Clone,” and the second report of the pandemic HA-MRSA lineage ST5/ST225-MRSA-II (“Rhine-Hesse EMRSA/New York-Japan Clone”) in the GCC, following its initial identification in Kuwait (Boswihi et al., 2016). Variants of the pandemic CC8-MRSA-[IVa + ACME I] (PVL+) “USA300” strain were also detected. Other pandemic MRSA clones detected included CC30-MRSA-IV (PVL+) (“Southwest Pacific Clone”) and the HA-MRSA lineage CC239-MRSA-[*mec* III + *Cd/Hg* + *ccrC*] (Senok et al., 2020). These clones are of particular concern as they are characterized by enhanced adaptability and virulence (Senok et al., 2020).

The study also reported the first detection of PVL-positive CC398 MRSA in the GCC region, a strain originally identified in South-East Asia (Moller et al., 2019). Its presence in the UAE suggests a likely introduction from this geographic region. Additionally, the first identification of the CC5-MRSA-IV “Sri Lanka Clone” within the country was documented (Senok et al., 2020). These findings emphasize the critical importance of ongoing genomic surveillance to monitor the emergence and spread of novel MRSA strains in the region.

A concerning upward trend in fusidic acid resistance, mediated by the SCC*fusC* genetic element was reported. It was found that 28.9% of the analyzed strains carried this resistance determinant (Senok et al., 2020). This trend calls for further investigation into the underlying factors contributing to the increased resistance, with community misuse of fusidic acid being one of the proposed drivers. Another critical finding from this study was the increase in prevalence of the *pvl* genes detected in 49% of isolates under investigation (Senok et al., 2020). This high percentage of PVL-positive strains in the country was further illustrated in another UAE study, in which 57 out of 187 MRSA isolates identified were classified as CA-MRSA and harbored *pvl* (Dash et al., 2014). This is particularly significant, as PVL-positive strains are associated with increased virulence, enabling them to penetrate intact skin and cause more severe infections (Gulmez et al., 2012).

A more recent study of MRSA recovered in the UAE in 2022 was also reflective of the Senok et al. (2020) study. This study involved genomic profiling of 310 clinical MRSA strains and assigned isolates to 22 clonal complexes and 72 distinct strain types, revealing predominance of CC1, CC5, CC6, CC8, CC22, and CC361 (Boucherabine et al., 2025). The study reported the first encounter of PVL-negative CC772-MRSA-V/VT in the UAE, in addition to a novel CC361-MRSA-IV (tst1+/PVL+) variant. In addition, upward trends of CC1153 were observed, alongside other rare CCs, such as CC121-MRSA and CC7-MRSA, marking the first documented report of the latter in region. Similarly, the CC5-MRSA-IV “Pediatric/WA MRSA-74 Clone,” which was previously unreported in the UAE was identified in this study. Along with the continued prevalence of PVL-positive CC5-MRSA-[IV + *fusC* + *ccrAB*] (“Maltese Clone”) and the ST5/ST225-MRSA-II (“Rhine-Hesse EMRSA/New York-Japan Clone”), these findings point to an ongoing expansion of the CC5-MRSA lineage within the UAE healthcare system. They also reflect a broader shift in the dominant circulating MRSA strain types in the region. Clonal complexes that were previously predominant in the UAE, such as CC80, CC97, CC239, and CC779 were detected in smaller numbers in this study. The *pvl* genes were detected in 38% of the study isolates, predominantly associated with skin and soft tissue infections, while the SCC-encoded fusidic acid resistance gene (*fusC*) was identified in 14% of isolates. Unsurprisingly, CA-MRSA lineages were also dominant in this study, with only one HA-MRSA isolate identified (Boucherabine et al., 2025).

Another noteworthy observation is the shifting epidemiology of CA-MRSA acquisition. Despite its designation as a community-associated pathogen, a study conducted at a tertiary care hospital revealed that only one-third of identified CA-MRSA cases were truly acquired in the community (Sonnevend et al., 2012). This finding highlights the successful introduction and dissemination of CA-MRSA clones within the hospital environment (Sonnevend et al., 2012).

Notably, a recent study was the first to document the presence of MRSA in the UAE food chain. *S. aureus* was found in 4.6% (14/343) of the salad samples that were analyzed (Habib et al., 2024). One of the isolates recovered from freshly imported dill was identified as MRSA, belonging to ST672 and *spa* type t384. This finding highlights the role of imported produce in the introduction of MRSA into regional food supplies and as a potential mode of dissemination (Habib et al., 2024).

### 3.2 MRSA in Saudi Arabia

Within Saudi Arabia, MRSA presents a complex epidemiological picture marked by wide clonal diversity, continuously evolving antimicrobial resistance patterns, and shifting dynamics between HA- and CA-MRSA. Numerous studies indicate growing predominance of CA-MRSA in Saudi Arabia over time (Alhejaili et al., 2025; Khanfar et al., 2012; Alsubaie et al., 2012; Alanazi et al., 2025). For instance, a retrospective analysis of 878 MRSA cases reported that CA-MRSA accounted for 88.4% of infections, with HA-MRSA comprising only 11.5% (Khanfar et al., 2012). This shift was observed alongside a decrease in nosocomial MRSA infection rates, possibly linked to stricter infection control policies and early screening protocols (Khanfar et al., 2012).

Molecular epidemiological findings across the country revealed that SCC*mec* type IV remains predominant, particularly among CA-MRSA strains, while HA-MRSA isolates are more likely to carry types I to III (Alhejaili et al., 2025). The co-circulation of both lineages within hospital settings has also been reported, with notable overlap in their resistance profiles (Mazi et al., 2020). In one study comprising of 102 isolates, CC80 (PVL+), CC22, and CC5 were among the most frequently detected clonal complexes, with *pvl* genes prevalence ranging from 30 to 50%, and in some instances, even present in HA-MRSA strains (Monecke et al., 2012). Recent studies have expanded the known molecular landscape of MRSA in Saudi Arabia by identifying both established and novel clones (Alarawi et al., 2025; Alhejaili et al., 2025). ST239-MRSA-III, once dominant in healthcare settings, continues to circulate (Monecke et al., 2018), though it appears to be gradually replaced by more diverse community-associated clones such as CC22-MRSA-IV and ST80-MRSA-IV (Senok et al., 2019b). A particularly notable finding is the first report of CC361-MRSA [V/VT + *fus*] in Saudi Arabia, described in isolates from the eastern region and genetically similar to strains previously identified in Kuwait indicating potential cross-border MRSA transmission within the Gulf region (Senok et al., 2019b). Other emerging clones include CC6, CC88, and ST97, some of which have been detected in both hospital and community settings, often exhibiting multi-drug resistance and a PVL-positive profile (Senok et al., 2019b). These findings emphasize the increasing complexity of MRSA epidemiology in KSA, with regional clone expansion and local adaptation shaping the current strain dynamics.

Importantly, a recent study in Saudi Arabia represents the most comprehensive genomic analysis of MRSA epidemiology in the GCC to date (Alhejaili et al., 2025), revealing the expansion and ongoing evolution of CA-MRSA clones and providing a critical reference for clonal epidemiology in the region. This large-scale nationwide analysis included systematically collected *S. aureus* isolates from 34 Ministry of Health hospitals across all provinces between 2022 and 2024, comprising 581 MRSA and 31 methicillin-susceptible *S. aureus* (MSSA) isolates from diverse body sites (Alhejaili et al., 2025). Short- and long-read whole-genome sequencing, combined with phylogenetic and population genomics analyses, enabled high-resolution characterization of population diversity and identified both globally distributed and regionally emerging lineages. The study also documented the rapid expansion of dominant clones, such as a subclade of the PVL-positive USA300/ST8-MRSA, estimated to have emerged within the past 15 years (Alhejaili et al., 2025). The MRSA population in this study exhibited substantial diversity (Alhejaili et al., 2025), comprising 48 distinct STs, most carrying community-associated SCC*mec* loci (types IVa, V/VII, and VI). Nine STs were novel and assigned new identifiers. Virulence factors associated with CA-MRSA, including PVL, were detected in 12 STs. Despite this diversity, a few clones predominated, notably ST8 (*n* = 110), ST5 (*n* = 63), ST88 (*n* = 56), ST6 (*n* = 52), ST672 (*n* = 47), ST30 (*n* = 46), ST97 (*n* = 35), ST22 (*n* = 30), and ST152 (*n* = 30), collectively accounting for 75% of isolates. The dominant clones which included ST8-t008 (USA300), ST6-t304 (CC6), ST88-t690, ST672-t3841 (CC361), and ST5-t311 were widely disseminated, and were associated with infections at multiple body sites. Interestingly, certain clones demonstrated site-specific associations; ST5, ST97, and ST8 were more frequently linked to bloodstream infections, whereas ST6 and ST152 were predominantly associated with wounds. Notably, ST152 was not detected in blood samples. The genomic analysis revealed extensive resistance determinants, including widespread *fusC* carriage, frequent aminoglycoside resistance genes (*aadD, aphA3, ant(4′)-Ia*), fluoroquinolone-resistance–conferring mutations, and composite SCC*mec*-*fusC* elements, reflecting selective pressure from fusidic acid use (Alhejaili et al., 2025). Virulence profiling showed high PVL prevalence, particularly in CC80 and CC22 lineages, with subsets of isolates carrying enterotoxin genes (*sea, seb, sek, seq*) and the toxic shock syndrome toxin (*tst*). Furthermore, the study also employed a hybrid sequencing approach to characterize the complete plasmid content of the isolates (Alhejaili et al., 2025). The analysis revealed a diverse array of *blaZ*-carrying plasmids and the dissemination of *erm(C)*-encoding plasmids across major clades. These findings indicate that MRSA in Saudi Arabia is driven by both clonal expansion and horizontal acquisition of mobile genetic elements, with plasmid acquisition occurring alongside clonal expansion.

The antibiotic resistance profile of MRSA isolates in Saudi Arabia is continuously evolving, with persistent resistance observed particularly among HA-MRSA strains. Resistance to erythromycin, tetracycline, and clindamycin remains commonly reported. One study found resistance rates of 46% to fusidic acid, 39% to tetracycline, and 36% to ciprofloxacin, with certain strains exhibiting resistance to up to eight different antimicrobial agents (Mazi et al., 2020). The widespread, often unregulated, over-the-counter use of fusidic acid is suggested to be a significant contributor to the expansion of resistance among MRSA clones. Notably, composite SCC*mec*-SCC*fusC* elements have been increasingly detected in both clinical and foodborne isolates, including within novel variants such as CC5-MRSA-[VI + *fus+tirS*] and CC15-MRSA-[V + *fus*] (Senok et al., 2019b).

While vancomycin remains largely effective against *S. aureus*, recent surveillance data suggest concerning trends in reduced susceptibility. A study of 736 MRSA isolates demonstrated a gradual increase in vancomycin minimum inhibitory concentrations (MIC creep), indicating diminished susceptibility over time (Aljohani et al., 2020). A large-scale WGS study carried out across all provinces of KSA between 2022 and 2024 identified rare but clinically significant resistance determinants (Alhejaili et al., 2025). Mutations in the *vraR* gene (involved in cell-wall stress response) and the *murF* gene (involved in peptidoglycan biosynthesis) were detected in three isolates (ST8 and ST88), conferring intermediate resistance to vancomycin. The *cfr* gene, which mediates resistance to linezolid, was also detected (Alhejaili et al., 2025). Although resistance to linezolid remains uncommon, it is reported sporadically in KSA. A study at King Khalid University Hospital detected phenotypic linezolid resistance in 2.9% (11/371) and vancomycin resistance in 0.3% (1/371) of MRSA study isolates (Al-Sarar et al., 2024).

Alarmingly, vancomycin-resistant *S. aureus* (VRSA) strains carrying the *vanA* gene have been reported among hospitalized patients in the Al-Jouf province, primarily in wound infections (Almuhayawi et al., 2023). A recent study at Prince Mutaib Bin Abdulaziz Hospital and Swair General Hospital found that MRSA accounted for 65% (123/188) of *S. aureus* wound infections, while 2.7% (5/188) were classified as *vanA* positive VRSA (Almuhayawi et al., 2023). Although these cases remain uncommon, the emergence of linezolid- and vancomycin-resistant *S. aureus* strains is a serious concern, given the reliance on these agents for treating MRSA infections. These findings highlight the growing emergence of multidrug-resistant clones in the region.

In addition to public health concerns within clinical settings, Saudi Arabia also faces the added challenge of zoonotic and foodborne transmission of MRSA. The pathogen is increasingly isolated from retail food products and livestock; including raw camel meat, dairy products, milk and goats, with several genotypes closely resembling those found in human clinical strains (El-Deeb et al., 2022). Notable strains associated with potential foodborne transmission include ST5, ST6, ST80, and CC15, often carrying SCC*mec* types IV/V and harboring key virulence and resistance determinants such as *fusC* and *pvl* (El-Deeb et al., 2022; Raji et al., 2016; Alghizzi and Shami, 2021). These findings support a One Health approach to MRSA surveillance, extending beyond hospital boundaries.

Collectively, the MRSA landscape in Saudi Arabia mirrors both global and regional trends, whereby novel hybrid lineages are rapidly emerging and previously predominant HA-MRSA clones are now frequently displaced by highly successful CA-MRSA clones exhibiting increased multi-drug resistance. These findings emphasize the urgent critical need for coordinated national MRSA surveillance programs, robust antibiotic stewardship, and stricter regulation of topical antimicrobials such as fusidic acid to curb further evolution and resistance.

### 3.3 MRSA in Kuwait

Antibiotic resistance remains a persistent ongoing public health concern in Kuwaiti hospitals. Numerous studies have documented rising resistance rates to antibiotics such as kanamycin, tetracycline, gentamicin, ciprofloxacin, and erythromycin/clindamycin (Alfouzan et al., 2023; Boswihi et al., 2016; Udo and Boswihi, 2017; Sarkhoo et al., 2021). In contrast, high-level resistance to mupirocin has shown a notable decline in recent years (Udo and Boswihi, 2017). Additionally, resistance to fusidic acid has also been increasingly documented, primarily mediated by the *fusC* gene. In one study, resistance was observed in over 80% of tested isolates, with other contributing resistance determinants including mutations in *fusA* and presence of the *fusB* gene (Boloki et al., 2021). It has been suggested that this increase may be driven by the extensive widespread over-the-counter use of topical antibiotic creams that contain fusidic acid, which are readily available without the need for a prescription (Boswihi et al., 2020). This underscores the urgent need for regulatory oversight and antimicrobial stewardship in the GCC region.

The MRSA landscape in Kuwait is marked by a high prevalence of both HA-MRSA and CA-MRSA, with recent shifts indicating a growing dominance of community clones, like other GCC countries. A large retrospective study analyzing 6,922 MRSA isolates from 2011 to 2015 reported that CA-MRSA accounted for 60.7% of isolates, while HA-MRSA made up 38.1% (Udo and Boswihi, 2017). This epidemiological shift is also reflected in the SCC*mec* distribution, with SCC*mec*-IV being the most prevalent type (53.9%), followed by types III and V (28.8 and 14.8%, respectively) (Udo and Boswihi, 2017).

Molecular characterization of MRSA in Kuwait shows that, unlike in the UAE and KSA, the historically dominant CC8/ST239-MRSA-III strain continues to persist (Boswihi et al., 2016). Recent studies have also reported an increasing prevalence of MRSA strains belonging to clonal complexes CC5, CC6, CC22, and CC97, often associated with SCC*mec* types IV, V, and VI (Boswihi et al., 2018; Boswihi et al., 2016; Alfouzan et al., 2019). In one study, a total of 400 non-duplicate MRSA isolates collected from 13 public hospitals in Kuwait between 1992 and 2010 were analyzed (Boswihi et al., 2016). Molecular typing identified 31 MRSA clones, with ST239-MRSA-III being the most prevalent (52.2%), followed by ST22-MRSA-IV (9.2%), ST80-MRSA-IV (7.5%), ST5-MRSA-II/IV/V/VI (6.5%), ST30-MRSA-IV (3.5%), ST241-MRSA-III (2.7%), ST6-MRSA-IV (2.2%), ST36-MRSA-II (2%) and ST772-MRSA-V (1.75%). SCC*mec* typing revealed five types, predominantly type III (55.2%) and type IV (31.7%). Among 60 identified *spa* types, the most common were t421 (21.5%) and t037 (19%), with other frequently detected types including t945, t044, t223, and t860 (Boswihi et al., 2016). In addition, 15.5% of the MRSA isolates were PVL-positive. The prevalence of PVL-positive MRSA in Kuwait has historically been high, with a 2013 study reporting *pvl* genes detection in 45% of isolates (Vali et al., 2017).

A 2016 study by Alfouzan *et al*., reported a prevalence of 30.8% for PVL-positive MRSA isolates (Alfouzan et al., 2019). A follow-up study by the same authors in 2020 (Alfouzan et al., 2023), conducted within the same hospital setting, revealed a notable decrease in this prevalence to 21.7%. The 2020 genotyping analysis provided updated insights into antibiotic resistance patterns and shifts in the clonal composition of MRSA within the Kuwaiti hospital, most notably documenting the disappearance of the previously dominant ST239-MRSA-III clone. This study included 60 MRSA isolates recovered from Farwaniya Hospital, a major public general hospital in Kuwait. Isolates were classified into 30 spa types and 13 CCs (Alfouzan et al., 2023). The most prevalent spa types were t304, t442, t311, t688, and t1234, which together accounted for 28.3% of the isolates. Clonal complexes CC5 (e.g., CC5-MRSA-V + SCC*fus*, CC5-MRSA-VI + SCC*fus*; CC5-MRSA-IV [PVL+]; CC5-MRSA-IV, “Pediatric Clone [*edinA*+]”) and CC6 (e.g., CC6-MRSA-IV and CC6-MRSA-IV + V,) were the most dominant, comprising 46.7% of the total isolates, followed by CC15 (CC15-MRSA-V + SCC*fus*), CC22 (CC22-MRSA-IV [tst1+], UK-EMRSA-15/Middle Eastern variant & a dual SCC*mec* IV + V type) and CC97 (CC97-MRSA-V [*fusC*+] and CC97-MRSA-IV) (Alfouzan et al., 2023). Other clonal complexes included CC1, CC8, CC30, CC80, CC88, CC121, CC152, and CC361.

All isolates were susceptible to vancomycin, rifampicin, linezolid, teicoplanin and mupirocin, although there were reported increases in the MICs of vancomycin and teicoplanin compared to 2016 (Alfouzan et al., 2023; Alfouzan et al., 2019). Resistance to fusidic acid (72%), ciprofloxacin (51.7%), trimethoprim (45%) remained prevalent, consistent with findings from the 2016 study. In contrast, the proportion of strains resistant to kanamycin (33.3%) and gentamicin (23.3%) declined in 2020 relative to 2016, while resistance rates to erythromycin (31.6%) and inducible clindamycin (21.7%) remained largely unchanged.

SCC*mec* typing identified the presence of types V, IV, IV + V, and VI among the isolates (Alfouzan et al., 2023). All 60 study isolates harbored virulence genes related to adhesins, clumping factors A and B, hemolysins, leukocidins, and biofilm formation. Notably, the previously dominant ST239-MRSA-III clone, which prevailed in the hospital environment in 2016 and earlier, was completely absent in 2020, suggesting a potential epidemiological shift toward the emergence of CA-MRSA clones within this setting (Alfouzan et al., 2023; Alfouzan et al., 2019). Correspondingly, *spa* types t860 and t945, commonly linked to ST239-MRSA-III and dominant in the 2016 study, were also absent in 2020 (Alfouzan et al., 2023). However, a more recent study from 2024 indicates that despite global trends favoring CA-MRSA, the CC239 lineage continues to evolve and remains capable of causing outbreaks (Monecke et al., 2024). Two recently identified variants of CC239-MRSA-III acquired pathogenicity-associated genes from coagulase-negative staphylococci; one from Trinidad carrying *speG* and the zinc resistance *crzC* gene on an SCC*mec*-associated mobile genetic element, and another from Kuwait (Monecke et al., 2024). The CC239-MRSA strain circulating in Kuwait appears to have evolved from a local variant of the “South-East Asian Clade” of CC239-MRSA-III through acquisition of the *speG* + *ccrA/B-2* recombinase genes + E-domain protein *edcP*-SCC element (Monecke et al., 2024).

Multiple studies have reported the emergence of novel or previously uncommon MRSA clones (Udo et al., 2020; Sarkhoo et al., 2021; Udo and Al-Sweih, 2013). Notably, CC361-MRSA [V/VT + *fusC*] has shown rapid expansion across Kuwaiti hospitals, with ST672-MRSA emerging as the dominant genotype characterized by high fusidic acid resistance and absence of PVL (Sarkhoo et al., 2021). Similarly, the once-rare CC15-MRSA clone has recently gained prominence, exhibiting multidrug resistance and harboring multiple virulence-associated genes, including clumping factor genes (*clfA/clfB*), cell-wall-associated fibronectin-binding proteins (*ebh, fnbA/B*), and biofilm-associated genes, *icaA/icaC/icaD* (Udo et al., 2020). The emergence of novel MRSA lineages including SCC*mec*-IV ST60-t3935 and SCC*mec*-IV ST194-t6892 within maternity hospitals in Kuwait was reported in 2013 (Udo and Al-Sweih, 2013). Additionally, the CC121-MRSA clone which is rare compared to the globally prevalent CC121-MSSA has been identified in Kuwait and more recently in the UAE (Boucherabine et al., 2025; Alfouzan et al., 2019). These findings suggest persistence and potential regional dissemination of rare clones in the GCC region.

Reported data from Kuwait has also highlighted rising concerns regarding resistance to older antibiotics (Vali et al., 2017; Udo et al., 2021). A resurgence of chloramphenicol resistance was documented, linked to *fexA*-positive CC5 clones, indicating clonal dissemination rather than isolated spontaneous mutations (Udo et al., 2021). Similarly, reduced susceptibility to chlorhexidine has been observed, linked to the presence of *qac* genes or other resistance mechanisms (Vali et al., 2017). While vancomycin and linezolid remain effective against MRSA, a significant proportion of isolates exhibiting elevated vancomycin MICs (3 μg/mL), was recently reported (Almuhayawi et al., 2023), raising concerns about diminishing therapeutic efficacy and the potential for resistance development.

MRSA can colonize animals and food products, including poultry, cattle, dairy items and fresh produce thereby presenting a potential route for foodborne transmission to humans. Despite the large number of reports on clinical MRSA isolates in Kuwait there is a notable lack of data on MRSA from food and animal sources. Indeed, although data on MRSA in the food supply and animal stock exists in countries such as the UAE (Habib et al., 2024) and Saudi Arabia (El-Deeb et al., 2018), there remains a substantial paucity of data for other GCC countries including Kuwait. In the absence of targeted surveillance, this pathway remains an ongoing and unmonitored public health risk. This gap in knowledge significantly hinders the ability to assess the potential contribution of zoonotic and foodborne transmission pathways within the country. Addressing this limitation will require the expansion of surveillance activities beyond hospital settings to encompass community, veterinary, and food production environments.

The shifting molecular profile of MRSA in the GCC region, illustrated here by trends observed in Kuwait highlights the urgent need for sustained, systematic surveillance. Such efforts should be complemented by the implementation of robust antimicrobial stewardship programs, tailored to the unique epidemiological landscape of each country, to effectively mitigate the spread and public health impact of MRSA.

### 3.4 MRSA in Oman

Although MRSA has been reported in Oman since the mid-1990s (Udo et al., 2014; Prasanna and Thomas, 1998), genotypic data on the prevalent lineages and clones circulating in the country remain scarce. The first study to provide insight into the prevalence and molecular epidemiology of MRSA in Oman was published in 2014 and analyzed 79 non-duplicate clinical MRSA isolates collected in 2011 from a major tertiary teaching hospital at Sultan Qaboos University (Udo et al., 2014). Among the study isolates, 86.0% harbored SCC*mec* type IV, while 10.1% carried SCC*mec* type V (Udo et al., 2014). Only two isolates contained SCC*mec* type III, and a single isolate carried SCC*mec* type II; SCC*mec* type I was not detected. The isolates exhibited considerable genetic diversity, with a predominance of CA-MRSA clones (91.2%), notably ST6-IV/t304 (39.2%) and ST1295-IV/t690, followed by ST772-V/t657, ST30-IV/t019/t021, ST80-IV/t044 and ST5-IV/t002 (Udo et al., 2014). The dominance of ST6-IV/t304 during this period marked a notable contrast to the MRSA profiles reported in neighboring countries, where ST239-III-MRSA was predominant in Saudi Arabia and ST30-IV-MRSA in Qatar (El-Mahdy et al., 2014; Udo et al., 2014; Monecke et al., 2012). Interestingly, 93.3% of the dominant ST6-IV/t304 clone did not harbor *pvl* genes and were also susceptible to non-*β*-lactam antibiotics (Udo et al., 2014). A similar observation was noted in a hospital in the UAE prior to this (Udo et al., 2014; Sonnevend et al., 2012). These findings indicate potential expansion and regional circulation of this clone within the GCC. The HA-MRSA lineages identified in Oman included ST22-IV/t852, ST239-III/t632, ST5-III/t311, and ST5-II/t003. Interestingly, while ST22-IV-MRSA has been previously reported in numerous other GCC countries, the PVL-positive ST22-IV/t852 clones identified in this study closely resembled those described in Qatar but differed from the ST22-IV/t005 variant reported in the UAE at the time (El-Mahdy et al., 2014; Udo et al., 2014; Monecke et al., 2012). Furthermore, most of the ST22-IV/t852 isolates identified were MDR strains, in contrast to the PVL-negative, non-MDR ST22-IV strains previously reported in Kuwait and the UAE (Udo et al., 2014; Sonnevend et al., 2012; Udo et al., 2008). These findings suggest that ST22-IV/t852 may represent an emerging multidrug-resistant variant of the ST22-IV MRSA lineage in Oman.

Additionally, 44.3% (35/79) of the MRSA isolates in this report from Oman carried the *pvl* genes (Udo et al., 2014), a proportion higher than the 14.6% reported in Kuwait at the time (Udo et al., 2014; Alfouzan et al., 2013), but lower than the 54.2% observed in a Saudi Arabian hospital (Udo et al., 2014; Monecke et al., 2012), highlighting the regional variability in the distribution of PVL-positive MRSA strains within GCC countries. All the study isolates were susceptible to vancomycin, teicoplanin, linezolid, tigecycline, and mupirocin. Resistance was observed to tetracycline, erythromycin, clindamycin, trimethoprim, ciprofloxacin, fusidic acid and gentamicin. One isolate showed chloramphenicol resistance. All were resistant to mercuric chloride, and 86.1% to ethidium bromide and cadmium acetate. 11.3% of isolates were classified as MDR strains (Udo et al., 2014).

### 3.5 MRSA in Qatar

Within the GCC region, there remains a significantly larger gap in epidemiological data on MRSA for certain countries, including Qatar. A study conducted in 2013 provided one of the first insights into the molecular epidemiology and resistance patterns of MRSA in the country (El-Mahdy et al., 2014). This study characterized MRSA isolates from multinational patients collected over a two-year period (2009–2010) from a private 250-bed tertiary-care general hospital in Doha, Qatar. Most of the study isolates were identified as CA-MRSA strains, including PVL-positive USA1100/ST30-MRSA (“Southwest Pacific Clone”) as the most prevalent clone representing 28% of the total number of isolates (El-Mahdy et al., 2014). Other well-established CA-MRSA strains such as ST80-MRSA, USA300/ST8-MRSA-IV and USA400/ST1-MRSA were also among the prevalent clones identified. The remaining isolates were well-known HA-MRSA clones including EMRSA-15/ST22-MRSA and the USA800/ST5-MRSA “Pediatric Clone.” Eight percent of the isolates exhibited mupirocin resistance. According to the hospital’s infection control policy, mupirocin nasal ointment was routinely employed for MRSA decolonization in carriers and for patient treatment. The study’s finding of a high prevalence of CA-MRSA aligns with regional and global epidemiological trends (El-Mahdy et al., 2014).

### 3.6 MRSA in Bahrain

A review of available literature revealed a significant gap in the molecular characterization of MRSA in Bahrain. Nonetheless, despite limited local data, the growing prevalence of CA-MRSA and upward trends in PVL-producing MRSA observed across the GCC is also evident in Bahrain (Alsaleh et al., 2023). This was illustrated in a recent study examining the prevalence, AMR profile, and molecular characteristics of MRSA within a tertiary care facility in the country. In this study, 88% of identified MRSA isolates were characterized as CA-MRSA strains (Alsaleh et al., 2023). Among these MRSA isolates, 22% were MDR, which expressed resistance against agents like quinolones, macrolides, and folate pathway antagonists. SCC*mec* typing reflected the broader molecular epidemiological trend observed in the GCC region, with SCC*mec* types IV and V predominating in CA-MRSA and types II and III in HA-MRSA.

Unexpectedly, *pvl* genes were detected in 66% of CA-MRSA isolates and 33% of HA-MRSA isolates. This prevalence of *pvl* in Bahrain is considerably higher than the rates reported in other GCC countries (El-Mahdy et al., 2014; Alkharsah et al., 2018; Udo and Al-Sweih, 2017), which suggests a distinct epidemiological pattern within the country. This disparity may reflect differences in strain circulation, community transmission dynamics, or variations in sampling methodologies. To accurately identify the factors driving this trend, a larger and more comprehensive molecular characterization of MRSA strains circulating in Bahrain is essential. Furthermore, this disparity highlights the possible heterogeneity of MRSA epidemiology within the GCC region, emphasizing the urgent need for more region-specific data to help guide public health interventions.

## 4 Discussion

The burden of infections caused by MRSA remains a substantial public health concern, both globally and within the GCC region. Over the past two decades, the MRSA landscape has undergone significant genetic and epidemiological shifts. Addressing this evolving threat requires a comprehensive understanding of the molecular, clinical, and environmental determinants driving its epidemiology. Such insights are critical for informing the design and implementation of targeted, national AMR interventions, and mitigating the wider societal and economic impacts of MRSA.

The molecular epidemiology of MRSA in the GCC region is marked by a diverse and distinctive genomic landscape. Factors such as wide clonal diversity, a high prevalence of PVL-positive strains, and the predominance of CA-MRSA lineages harboring SCC*mec* types IV/V contribute to the evolving epidemiology of MRSA. Established clonal complexes of MRSA, including CC5, CC6, CC22, and CC30, continue to circulate and expand across the region, while variant strains, such as CC772-MRSA-V (PVL+, “Bengal Bay Clone”), CC5-MRSA-[V/VT + *fusC*], ST5/ST225-MRSA-II “Rhine-Hesse EMRSA/New York–Japan” clone, have also emerged (Senok et al., 2020; Boucherabine et al., 2025; Alhejaili et al., 2025), adding further complexity to the regional epidemiological landscape.

The shift in predominance from traditional SCC*mec* types I–III to IV/V, likely reflects multiple epidemiological and evolutionary factors (Alhejaili et al., 2025; Deurenberg et al., 2007). This trend suggests that CA-MRSA strains which typically harbor SCC*mec* IV/V are increasingly infiltrating healthcare settings, possibly facilitated by population mobility, healthcare interconnectedness, and evolving antibiotic selective pressures (Macpherson et al., 2009). These smaller SCC*mec* elements confer metabolic advantages, increasing both genetic fitness and transmissibility relative to the larger I–III elements (Alhejaili et al., 2025; De Miranda et al., 2007; Collins et al., 2010). For example, Alhejaili *et al.,* 2025 reported that clones carrying smaller SCC*me*c elements, along with quinolone resistance-determining regions, harbored higher virulence and resistance gene content (Alhejaili et al., 2025).

The proliferation of the composite SCC*mec*/SCC*fusC* element, which confers resistance to *β*-lactam antibiotics and to fusidic acid (Senok et al., 2019a; Ellington et al., 2015), a widely used topical antimicrobial agent in the GCC is also a concern (Al-Saleh et al., 2022). In addition to resistance determinants, there is a growing prevalence of key virulence factors among MRSA isolates in the GCC. These include genes encoding enterotoxins, epidermal cell differentiation inhibitors (*edin*), toxic shock syndrome toxin (*tsst-1*), and various biofilm-associated and adhesion-associated genes (Boucherabine et al., 2025). The increased detection of these resistance and virulence markers is likely a consequence of sustained selection pressure driven by widespread and often inappropriate use of antibiotics, which is a well-documented concern in the GCC (Al-Saleh et al., 2022; Senok et al., 2019a; Asokan et al., 2019). Over-the-counter access to antimicrobials, lack of enforcement of prescribing regulations, and inconsistent infection control practices may be facilitating the persistence and spread of virulent, highly drug-resistant MRSA strains in both healthcare and community settings. Together, these trends highlight the need to reassess the operational definition of MRSA in the region (Al-Saleh et al., 2022). Such redefinition would facilitate a more accurate understanding of its evolution, patterns of dissemination, and resistance mechanisms.

Notably, despite geographic proximity and frequent movement of national and expatriate populations across the GCC, countries in the region showed differences in the CCs/STs, AMR and virulence profiles of circulating strains. These differences likely result from a complex interplay of regional factors, including variations in healthcare infrastructure, infection control practices, and antimicrobial stewardship policies, which collectively create distinct selective pressures shaping local MRSA populations. High population mobility and density, especially in major urban centers and sectors such as healthcare, construction, and domestic work may facilitate the introduction and circulation of novel strains in capital cities (Neiderud, 2015). Larger tertiary hospitals often have formal stewardship teams, greater diagnostic capacity, routine surveillance, and reporting mechanisms, whereas smaller facilities may lack structured programs (Aika and Enato, 2022). Patient referral patterns, with complex or chronic cases concentrated in major hospitals, may further skew observed burdens (Donker et al., 2010).

Variations in antimicrobial prescribing practices across hospitals, private clinics, and community pharmacies may also contribute to regional differences in resistance (Alhomoud et al., 2017; Al-Mehmadi et al., 2023). In communities with inappropriate antibiotic use, including over-the-counter availability of topical agents such as fusidic acid, selective pressure may favor resistance elements such as *fusC* (Senok et al., 2019a). Although legislation prohibiting non-prescription antibiotic sales has existed in the region, enforcement was initially weak, allowing continued non-prescription use (Alrasheedy et al., 2020). Recent stricter measures have substantially reduced this practice, and ongoing implementation across the GCC is critical to curb antimicrobial resistance and preserve the effectiveness of existing therapies (Alrasheedy et al., 2020).

Finally, although data remain limited, regional differences in veterinary and agricultural antimicrobial practices may contribute to the presence of LA-MRSA strains in certain areas (Crespo-Piazuelo and Lawlor, 2021). Taken together, these multifactorial demographic, clinical, and environmental drivers likely underpin the observed diversity in MRSA epidemiology across GCC countries.

The detection of reduced susceptibility and emerging resistance to last-line agents, particularly vancomycin and linezolid, also highlights the growing AMR challenge in the GCC. Current guidelines recommend maintaining vancomycin trough concentrations above 10 mg/L to prevent resistance, with a 15–20 mg/L therapeutic range to minimize toxicity (Al-Maqbali et al., 2022). In a 2022 study from Oman, 16.8% (17/101) of patients failed to reach therapeutic levels, while 47.5% (48/101) exceeded 20 mg/L, emphasizing the need for stringent vancomycin therapeutic drug monitoring (TDM) (Al-Maqbali et al., 2022). Similarly, surveillance data from Saudi Arabia have documented vancomycin MIC creep and the presence of genetic determinants conferring linezolid resistance (Aljohani et al., 2020; Alhejaili et al., 2025). Although the overall prevalence remains low, VRSA have also been reported (Al-Sarar et al., 2024; Almuhayawi et al., 2023). These observations highlight the importance of routine TDM, MIC trend monitoring, mandatory reporting of resistant isolates, and continued investment in diagnostic capacity to safeguard last-line therapies and guide empiric treatment practices. Antimicrobial stewardship programs and infection control policies are increasingly being implemented across GCC healthcare facilities to mitigate the spread of AMR pathogens (Alrasheedy et al., 2020; Ababneh et al., 2021; Haseeb et al., 2023; Torumkuney et al., 2022; Faryal et al., 2023), focusing on optimizing antibiotic use and restricting broad-spectrum agents (Haseeb et al., 2023; Torumkuney et al., 2022; Rizk et al., 2021). However, progress varies across countries, further reflecting variability in infrastructure and resources (Torumkuney et al., 2022; Hashad et al., 2023; Haseeb et al., 2020).

The primary limitation identified in the molecular epidemiology of MRSA within the GCC, as highlighted by this review, is the fragmented and incomplete nature of existing research in the region. Of the 97 studies included, more than half were conducted in Saudi Arabia (58%), roughly a quarter in Kuwait (26%), and a smaller proportion in the UAE (7%), while Bahrain, Oman, and Qatar each contributed only a minimal fraction of the data (2–3%). This imbalance likely reflects disparities in research capacity, surveillance infrastructure, and publication output among GCC countries, rather than true differences in MRSA burden. Consequently, the findings of this review may more accurately represent the epidemiological situation in Saudi Arabia and Kuwait, with limited generalizability to the other GCC states. Furthermore, much of the available data derives from single-center or institution-specific studies, providing only cross-sectional snapshots rather than longitudinal or nationally representative insights into MRSA burden and trends. These limitations, coupled with significant gaps in the literature, hinder the development of a comprehensive understanding of MRSA transmission dynamics, resistance mechanisms, and clonal evolution across this geographically and demographically diverse region. To overcome these challenges, future efforts should prioritize region-wide collaborative surveillance, and greater inclusion of under-represented countries. Such initiatives are essential to generate a more complete and representative understanding of MRSA epidemiology across the GCC.

A further limitation is the absence of genomic data in existing national MRSA surveillance systems across the GCC, as well as in non-GCC countries with epidemiological links to the region, such as South Asian countries, which contribute substantially to the GCC population (Ali and Cochrane, 2024). To address this gap, we strongly advocate for the integration of genomic data into routine MRSA surveillance efforts. Beyond the need for expanded geographical and institutional surveillance coverage, there is also a clear under-utilization of high-resolution molecular tools, particularly WGS, across the region. Compared to conventional molecular typing methods, WGS provides significantly greater resolution for key epidemiological applications, including phylogenetic analysis, outbreak investigation, and the detection of novel resistance and virulence determinants (Azarian et al., 2015).

The scarcity of region-wide WGS data makes it challenging to establish detailed phylogenetic relationships between MRSA lineages circulating in the GCC and those on other continents. Available genotyping data indicate that GCC MRSA populations reflect both the importation of major European and Asian lineages, as well as ongoing local evolution. Classic European clones, such as EMRSA-15/ST22, as well as other HA-MRSA lineages like ST5/ST225-MRSA-II “Rhine-Hesse EMRSA/New York–Japan” are increasingly detected across Saudi Arabia, Kuwait, and the UAE (Senok et al., 2020; Tabaja et al., 2021; Boswihi et al., 2016). Meanwhile, the Indian-subcontinent community clone ST772 “Bengal Bay clone” and ST80-MRSA, typically associated with North Africa, have been repeatedly identified in several GCC countries (Boucherabine et al., 2025; Alfouzan et al., 2023; Alhejaili et al., 2025; Udo and Sarkhoo, 2010), consistent with introduction via labor migration and travel. Beyond these imported strains, the presence of composite SCC*mec*-*fusC* elements provides further evidence of local diversification (Boucherabine et al., 2025; Alhejaili et al., 2025), although confirmation of distinct evolutionary lineages requires comprehensive phylogenetic profiling. Incorporating WGS into clinical and public health laboratories alongside national surveillance frameworks would substantially enhance the region’s ability to monitor MRSA evolution, identify emerging high-risk clones, and implement timely public health interventions (NIHR Global Health Research Unit on Genomic Surveillance of AMR, 2020).

To strengthen infection prevention control practices across the GCC, more systematic and coordinated surveillance strategies could be implemented (Albali et al., 2023). For instance, mandatory MRSA admission screening in all secondary and tertiary care hospitals, alongside the routine submission of a standardized proportion of isolates to national or regional reference laboratories for molecular typing, would significantly enhance data quality on MRSA prevalence, resistance mechanisms, and clonal distribution (Struelens et al., 2009). These strategies would complement existing stewardship and infection control efforts. An encouraging example of regional advancements in AMR monitoring is the recent expansion of national AMR surveillance infrastructure in the UAE. Over the past decade, the number of reporting sites increased significantly from 22 in 2010 to 317 in 2021, enabling the analysis of over 29,000 MRSA isolates (Thomsen et al., 2023). Such scaling of surveillance capacity has strengthened the ability to detect epidemiological trends and guide timely interventions. Sustaining and replicating such initiatives across neighboring countries will be critical toward controlling AMR and anticipating future trajectory.

**Figure 4:**
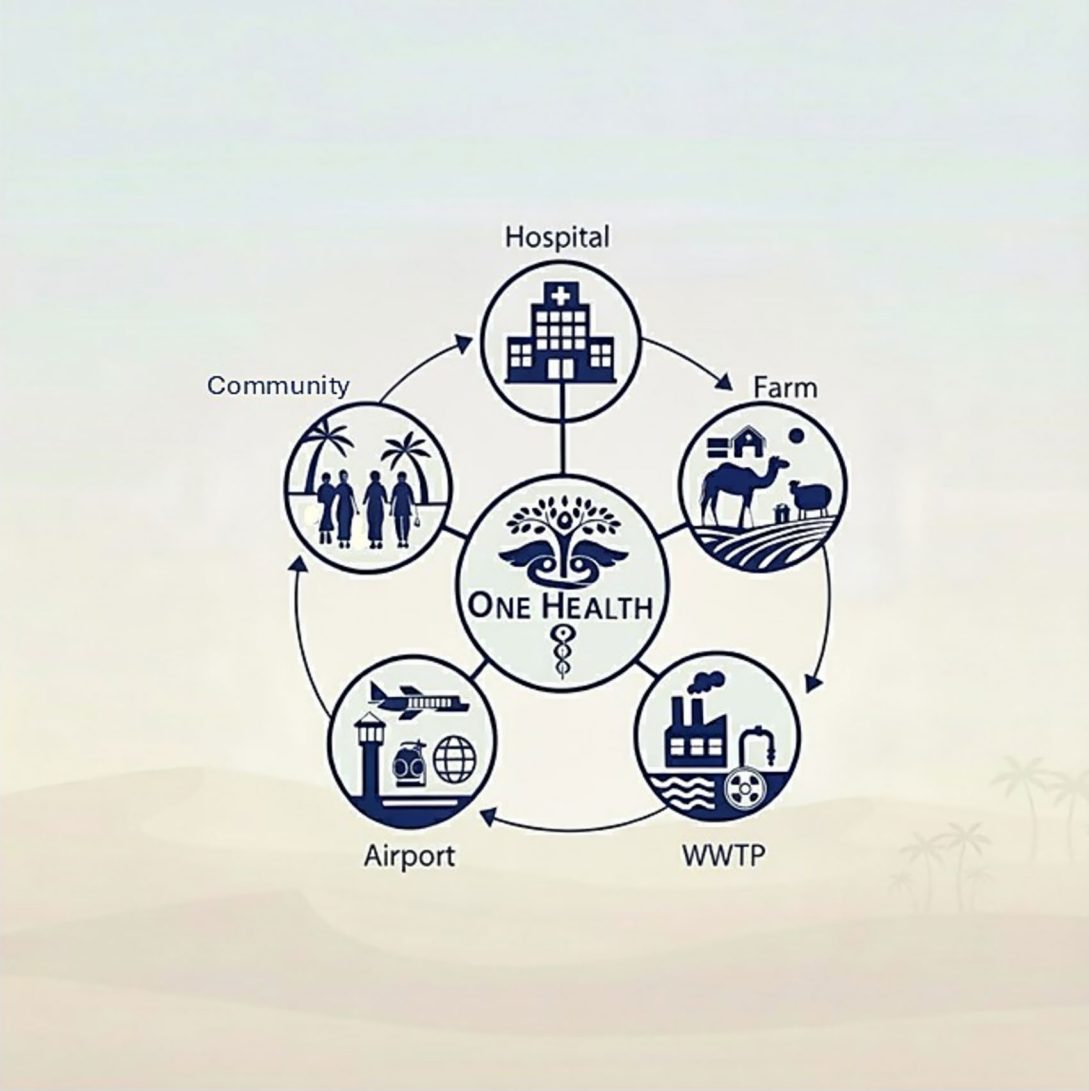
One Health approach for MRSA & antimicrobial resistance surveillance in the Gulf Cooperation Council (GCC) region. Highlights the interconnectedness between community, healthcare, international travel hubs (airports), livestock farms, and wastewater as key reservoirs. One Health recognizes the need for integrated, cross-sectoral surveillance to address the MRSA/AMR crisis. WWTP; wastewater treatment plant.

To enhance surveillance, the integration of a coordinated One Health approach into regional monitoring efforts is recommended (Figure 4). The One Health approach emphasizes the interconnectedness of human, animal, and environmental health, promoting a holistic strategy to investigate AMR/MRSA transmission dynamics (Aslam et al., 2021). Antimicrobial resistance is increasingly prevalent across all of these sectors due to contributing factors such as inappropriate antibiotic use, inadequate sanitation protocols, and ineffective infection control practices. By incorporating data from clinical, community, and livestock sources, alongside environmental sampling, this approach can provide deeper insights into the epidemiology of MRSA and inform more effective infection prevention and control strategies. Several countries have successfully adopted such integrated strategies (Norstrom et al., 2023; Moura et al., 2023; Grontvedt et al., 2016). For instance, Norway has implemented a national control process for livestock-associated MRSA (LA-MRSA) in swine, which includes comprehensive surveillance of the entire pig population and a targeted “search and destroy” policy to eradicate LA-MRSA from affected farms (Grontvedt et al., 2016). The underlying objective is to prevent pigs from becoming a persistent reservoir of MRSA, thereby reducing the risk of zoonotic transmission to humans. This model offers a valuable precedent for the GCC region in developing its own One Health-informed surveillance and control measures. To achieve this, the substantial gap in reported data on MRSA from animal and food sources in most GCC countries must first be addressed. This absence of data, both in the literature and within national surveillance systems, represents a critical limitation in understanding the full epidemiological scope of MRSA in the region and poses a significant barrier to the effective implementation of comprehensive One Health strategies. The gap in data is further compounded by the lack of standardized nomenclature and consistent strain definitions across countries, which hinders meaningful comparisons and limits the broader utility of existing findings. Establishing a unified nomenclature system in which all researchers use the same designation for a given strain would not only improve clarity in reporting but also facilitate cross-border comparisons, strengthen global communication, and ultimately enhance the effectiveness of regional infection control strategies.

Closely aligned with the principles of the One Health approach, wastewater-based surveillance (WBS) offers a promising and integrative tool for investigating AMR across human, animal, and environmental reservoirs (Figure 4). WBS is a valuable public health method that involves the systematic collection and analysis of wastewater samples, which can capture biological inputs from entire communities (Hendriksen et al., 2019). This approach, if implemented across the GCC would enable detection and analysis of evolving trends of pathogens such as MRSA at the population level, without the need for direct individual testing, thereby enhancing regional monitoring. This is particularly relevant given that hospital-based surveillance often underestimates the true burden of MRSA by excluding asymptomatic individuals or those who do not seek healthcare services. In contrast, WBS captures data from all contributors to the wastewater system, offering a more holistic view of pathogen circulation. Globally, WBS has demonstrated considerable utility in AMR surveillance. Countries such as the United States (National Academies of Sciences, Engineering, and Medicine, 2024), Sweden (Borjesson et al., 2009) and Finland (Tiwari et al., 2024) have successfully utilized WBS to detect and track resistant pathogens, contributing to timely public health responses. Recently, the European Union launched the EU Wastewater Surveillance Dashboard, a platform designed to integrate WBS data across member states, thereby providing early warnings for emerging infectious threats prior to widespread clinical detection (European Commission, 2025). While MRSA is not currently included in the EU surveillance dataset (European Commission, 2025), the existing infrastructure illustrates the feasibility of expanding WBS to include high-priority AMR pathogens. The adoption of WBS in the GCC could provide critical data to inform regional AMR policies and regulations. By complementing clinical and hospital-based surveillance systems, WBS offers a scalable and proactive approach to tracking MRSA and other resistant organisms.

## 5 Conclusion

In conclusion, this review highlights the urgent need for nationwide surveillance systems in the GCC, supported by standardized high-resolution sampling strategies and harmonized reporting frameworks. Strengthening surveillance capacity through such coordinated efforts will enable robust molecular characterization and a more accurate assessment of the true epidemiological burden of MRSA in the region. In addition, it will help close critical knowledge gaps in the literature and provide an essential foundation for more effective public health policies.
