# Correction: Molecular epidemiology of methicillin-resistant *Staphylococcus aureus* in Gulf Cooperation Council countries (2010–2025): a scoping review

**DOI:** 10.3389/fmicb.2026.1791202

**Published:** 2026-02-04

**Authors:** Frontiers Production Office

**Affiliations:** Frontiers Media SA, Lausanne, Switzerland

**Keywords:** *S. aureus*, methicillin-resistant, antimicrobial resistance, epidemiology, prevalence, GCC, Middle East

For reviewer Dalal Alkuraythi, the affiliation [University of Jeddah, Jeddah, Saudi Arabia] was erroneously given as [King Saud University, Saudi Arabia].

The original version of this article has been updated.

